# Effect of Bentonite Binder on the Critical Properties of Zeolite Extrudates in Dehumidification in Confined Space

**DOI:** 10.3390/ma19214741

**Published:** 2026-11-08

**Authors:** Pitcha Sawaiprakhon, Supansa Paipae, Jidapa Ouantavorn, Thanantorn Tong-Uthai, Juthathip Khanthawisooth, Chawisa Sae-Iah, Nichapach Suraged, Juthamas Sripitak, Supak Tontisirin

**Affiliations:** 1 Department of Chemical Engineering, Faculty of Engineering, King Mongkut’s University of Technology North Bangkok, Bangkok 10800, Thailand; phitcha.swp@gmail.com (P.S.); beenpaipaes@gmail.com (S.P.); jidapa.ouantavorn@gmail.com (J.O.); ttoon.juu@gmail.com (J.K.); chawisasaeiah@gmail.com (C.S.-I.); ketar_2345@hotmail.com (N.S.); jtmprang@gmail.com (J.S.); 2 Center of Eco-Materials and Cleaner Technology, King Mongkut’s University of Technology North Bangkok, Bangkok 10800, Thailand

**Keywords:** bentonite, binding agent, dehumidification, extrusion, zeolite shaping

## Abstract

**Highlights:**

- Cylindrical NaX zeolite extrudates prepared with bentonite binder exhibit a surface area of 556 m^2^/g, pore volume of 0.249 cm^3^/g, and radial crush strength of 43.19 N (4.8 N/mm).
- The optimal calcination of extrudates is achieved by heating at 3 °C/min to 600 °C with a 3 h holding time in air flow.
- Quartz, anorthoclase, and metabentonite can promote the mechanical stability of the zeolite extrudates.
- Bentonite decreases the surface area and pore volume of extrudates while creating mesopores (7.5–24 nm).
- An efficient thermal regeneration protocol for the extrudates is proposed.

**Abstract:**

The shaping of zeolite powder is an essential process for enhancing zeolite utilization. It facilitates easier handling, improves fluid flow distribution, minimizes pressure drop during operation, and enhances mass transfer. Adding a bentonite binder is necessary for zeolite particle cohesion to create strong bonds between the zeolite crystals. The resulting quartz, anorthoclase, and metabentonite phases can enhance the mechanical stability of the zeolite extrudates. In addition, the bentonite binder modifies the pore size distribution of the extrudates by generating mesopores in the 7.5–24 nm range. In contrast, adding more bentonite (26.7:100 bentonite-to-zeolite weight ratio) can reduce the specific surface area and pore volume by 35.9% and 32%, respectively. NaX zeolite extrudates are effective dehumidifying adsorbents for moisture control in confined spaces. Incorporating a bentonite binder into the extrudate creates a highly suitable hierarchical structure (mesopore-to-micropore ratio). This structure facilitates the diffusion of water vapor into the active sites within the extrudate core, thereby enhancing adsorption performance. The linear driving force (LDF) model combined with the Freundlich equation is empirically fitted to the adsorption kinetics of the zeolite extrudates. The extrudates remain stable for at least four cycles of post-regeneration with no loss in efficiency. These results demonstrate that the prepared NaX extrudates are suitable for this application and can be effectively utilized in large-scale applications.

## 1. Introduction

Dehumidification is a crucial process in food and pharmaceutical processing, storage, and environmental management. It maintains strict product quality and safety, prevents spoilage, and ensures indoor air quality and comfort by precisely controlling air humidity levels [1,2,3]. Regarding residential air quality control, humidity that is too high or too low can adversely affect living conditions. Consistent moisture control is also essential for laboratories and for protecting sensitive electronic equipment, specifically electronic packaging, which relies on a dry, inert atmosphere to safeguard delicate components.

Solid adsorption by zeolites is an effective method for dehumidification, as it traps water molecules within their crystalline structure [4,5]. Zeolites exhibit strong hydrophilic properties and higher adsorption capacities than classical silica gel, owing to their well-defined chemical structure and regular pore size. They work by trapping water vapor within their crystalline structure, making them ideal desiccants. They offer superior moisture adsorbents for a wide range of humidity levels, performing especially well in cool environments [6,7,8]. Due to their strong affinity for water vapor, they maintain high adsorption efficiency even at very low relative humidity.

For practical applications, zeolite powder must be formed into specific shapes. The extrusion process is one of the most versatile and simple shaping methods of zeolite powder [9]. The extrusion process involves forming shaped zeolites by forcing the material through a die to produce objects with a uniform cross-sectional profile. This process requires a binder to improve mechanical strength and a plasticizer to enhance the paste’s workability [10]. Typical binders are inorganic binders (kaolin, attapulgite, boehmite) and organic binders (polyvinyl alcohol (PVA), polyethylene glycol (PEG), and glycerin).

Bentonite, an inorganic binder, is a naturally occurring hydrous aluminosilicate clay powder. It has a sheet-silicate structure. It exhibits a high water adsorption capacity and undergoes significant swelling upon hydration [11]. The bentonite binder yields various zeolite structures with mechanical strengths in the range of 0.34–6.81 MPa [12,13,14,15,16]. Four preparation methods involving changing the sequence of preparation of Pt-modified Beta zeolite extrudates using bentonite binder (30%) affect the deposition of Pt in different locations in the extrudates. The in situ method with platinum primarily located on the zeolite exhibits the highest metal-to-acid-site ratio and the closest proximity. However, it has the lowest mechanical strength, but it shows acceptable mechanical strength. The mechanical strength can be correlated with the Pt loading (1.4–3.4%): the strength of the extrudate increases with increasing Pt concentration. The values lie between 3.25 and 4.8 MPa (5.01–7.39 N) [12]. The formation of SBA-15 mesoporous silica pellets with 20, 25, and 30% bentonite and 0, 5, and 10% colloidal silica binders showed compressive strengths of ca. 0.6–2.1 MPa (1.88–6.59 N). The colloidal silica provides a much lower contribution to mechanical strength compared to bentonite. The addition of 20% bentonite decreased specific surface area by 20% from 490 to 395 m^2^/g and pore volume by 15% [13]. NaA zeolite synthesized from coal fly ash was pelletized using bentonite and kaolinite as binders at contents of 5–10 wt.%. The kaolinite did not behave as an expected feature of a binder, whereas the bentonite supported the strength of the granules. The spherical granules (3–6 mm) prepared with 90 wt.% zeolite and 10 wt.% bentonite exhibited the highest fracture strength and breakage force at 2.9 MPa and 32.7 N, respectively [14]. ZSM-5 zeolites underwent a shaping process for monolith catalysts (10 mm diameter) using bentonite binder with a 3D-printed robocast. The binder concentrations were in the range of 40–60 wt.%. Increasing the bentonite binder concentrations, the compressive strengths increased from 0.34 to 1.61 MPa (26.71–126.5 N) (an increase by a factor of 4.5). Lower and higher bentonite:zeolite ratios negatively affect surface cracking and larger agglomeration, and sample shrinkage, respectively [15]. Binder types (silica sol, bentonite, attapulgite, and SB1) were studied for 13X zeolite shaping by applying only 20 wt.% to pristine zeolite. Among the extrudates, silica binder can make the strongest extrudate, and it is the most suitable binder for CO_2_ capture performance. However, the mechanical properties of the extrudates using bentonite were reported to be 6.51–6.81 MPa (20.5–21.4 N) [16].

The effect of binder on the properties of shaped zeolite depends sensitively on various parameters such as zeolite type, crystal size, morphology, formulation amount, and treatment conditions for shaping. Consequently, each binder–zeolite system exhibits unique characteristics. In this study, bentonite was selected as the binder due to its compositional similarity and hydrophilic nature, which align with the properties of NaX zeolite and its application. Systematic studies on how bentonite impacts the crystallographic, textural, and thermal properties of shaped NaX zeolites are scarcely reported in the literature. This might be confidential information in the industry. However, fundamental insights into these relationships are essential for successful zeolite shaping and for establishing a foundation for future research. This work aims to bridge this knowledge gap. Furthermore, a proper calcination program for the green extrudates and a regeneration procedure are proposed based on comprehensive observations.

The system of NaX zeolite shaping by extrusion utilizes bentonite as a binder, methylcellulose as a plasticizer, and water as a solvent. The amount of bentonite is varied, and the optimized composition is achieved to enhance the mechanical properties of the resulting zeolite extrudates while maintaining their essential performance characteristics. The zeolite extrudates will be characterized using X-ray diffraction (XRD), thermogravimetric analysis–differential scanning calorimetry (TGA-DSC), N_2_-adsorption, field-emission scanning electron microscopy (FE-SEM), and crush strength testing. Finally, the NaX zeolite extrudates will be evaluated for dehumidification in confined spaces, and their regeneration stability will be thoroughly investigated.

## 2. Materials and Methods

### 2.1. Preparation of Zeolite Extrudates

The NaX zeolite powder (Mizusawa, Tokyo, Japan) and distilled water were mixed and stirred by an overhead stirrer with a pitched-blade turbine. The bentonite (Acro, Geel, Belgium) was added to the mixture and stirred until well-mixed. Methylcellulose (Acro, Geel, Belgium) was added to the mixture and stirred until the paste became soft and thicker. Then, the paste was extruded by a handheld extruder equipped with a 2 mm circular die. The zeolite extrudates were cut with a sharp knife to a 10 mm length. The extrudates had a cylindrical shape, with dimensions of 2 mm in diameter and 10 mm in length. Thereafter, the extrudates were dried in a convection oven at 120 °C for 4 h and then calcined in a furnace at temperatures of 500–600 °C for 3 h in an air atmosphere at 0.6 L/min. Different concentrations of bentonite binder were used during shaping to study the impact of the binder on the key properties of the extrudates. The proportions of the components are presented in Table 1.

### 2.2. Characterization Methods

The NaX zeolite extrudates’ crystalline structure was investigated by XRD (Bruker, D2 Phaser, Karlsruhe, Germany) operating with a copper K-alpha X-ray source (λ = 1.5406 Å), at 30 kV, 10 mA, with an increment of 0.02 degrees, step time 0.6 s, and scan range of 2theta = 5–50. Surface areas and pore volumes were measured by low-temperature N_2_-adsorption (Micromeritics, ASAP 2460, Norcross, GA, United States) by applying the BET model, *t*-plot, and BJH analysis. The samples in extrudate form were preheated at 200 °C for 12 h before the measurement. The BET surface area was analyzed in the pressure range p/p_0_ ≈ 0.035–0.1. The micropore volume was determined by *t*-plot analysis in the thickness range of 3.5–5 Å. The appropriate calcination temperatures for the complete removal of organic compounds were investigated by analyzing the mass losses and thermal behavior of bentonite, methylcellulose, and the zeolite extrudates using TGA-DSC (Mettler Toledo, TGA/DSC 3+, Greifensee, Switzerland). The samples were tested by increasing the temperature from 25 to 800 °C with a heating rate of 10 °C/min in an air atmosphere. Mechanical properties were evaluated by radial crush testing (Shimadzu, EZ-S, Kyoto, Japan) at a head speed of 0.5 mm/min using a 500 N load cell. The samples, cut to a length of 9 mm, were placed horizontally on the lower plate and loaded until they fractured. The radial crush strength was determined. All tests were performed in triplicate.

### 2.3. Dehumidification in Confined Space Tests

**Figure 1:**
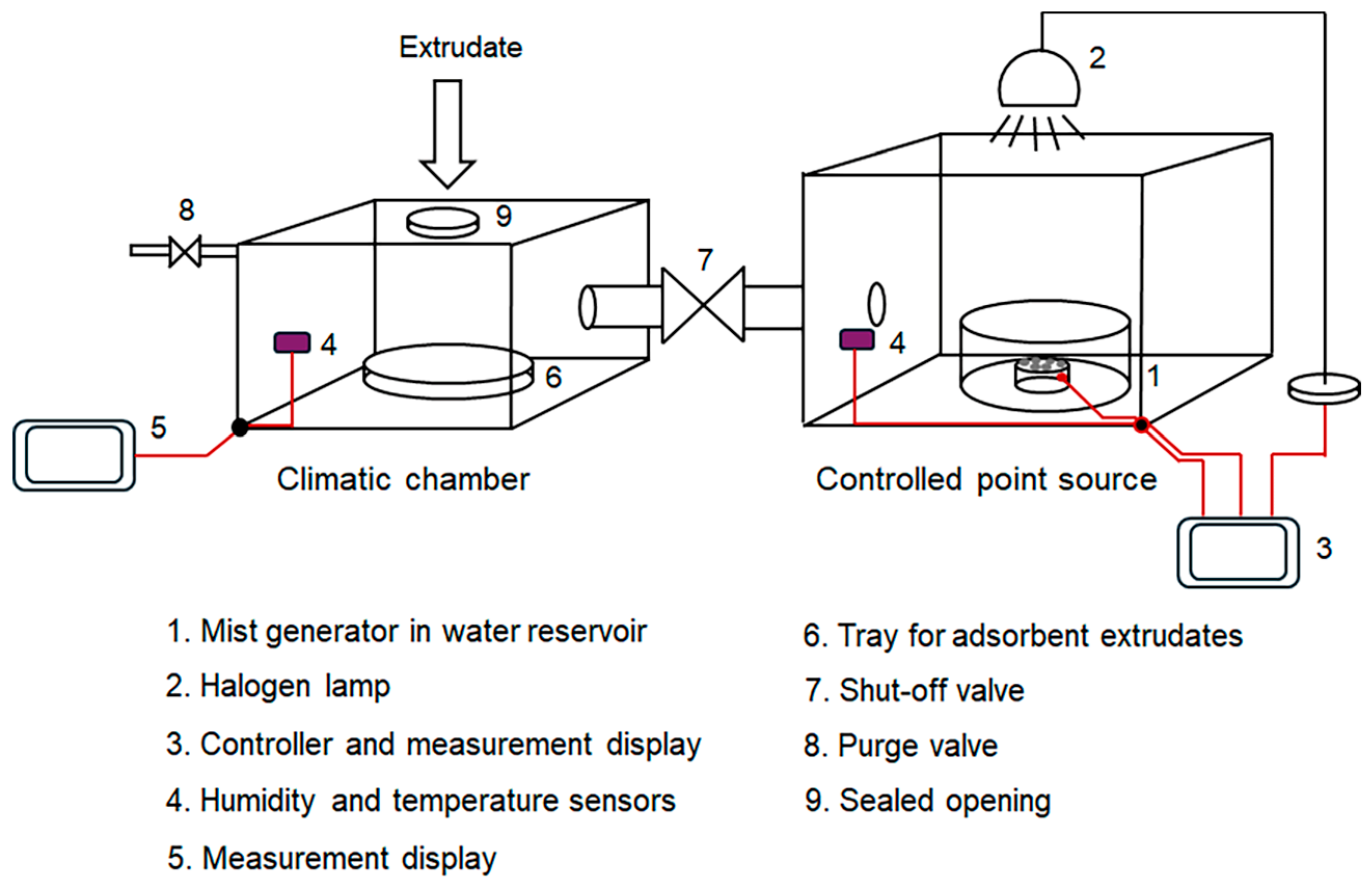
The diagram for the dehumidification experiment in a confined space.

Dehumidification tests using zeolite extrudates were conducted in a custom apparatus equipped with internal humidity and temperature sensors, and the wall was insulated. A climatic chamber was maintained at a relative humidity of 80% and a temperature of 27 °C. As illustrated in Figure 1, these conditions were established via a point source reservoir connected by a tube and valve system. Humidity was generated using a mist generator, while the temperature was regulated by a halogen lamp acting as a heat source. Once the target conditions were established, the point source was connected to the climatic chamber (volume of 1317 cm^3^) by opening the valve and purge tube. When the environments in both units equilibrated, the intervening valve and purge tube were closed. The candidate extrudates (37.7 g dry basis) were activated in a furnace at 350 °C for 3 h. Subsequently, it was cooled to room temperature within a sealed glass desiccator. Once the extrudates were introduced into the prepared climatic chamber, the dehumidification process began. The relative humidity was recorded for 30 min. The performance of dehumidification within the climatic chamber was evaluated based on the relative humidity profile. The sample was regenerated by calcining from room temperature to 350 °C at a heating rate of 3 °C/min with a holding time of 3 h in N_2_ flow at 0.6 L/min. Subsequently, it underwent a second cycle of dehumidification testing. This process was repeated for four cycles. The powder forms of bentonite and NaX with the same weight were also tested.

## 3. Results and Discussion

### 3.1. Material Preparation

**Figure 2:**
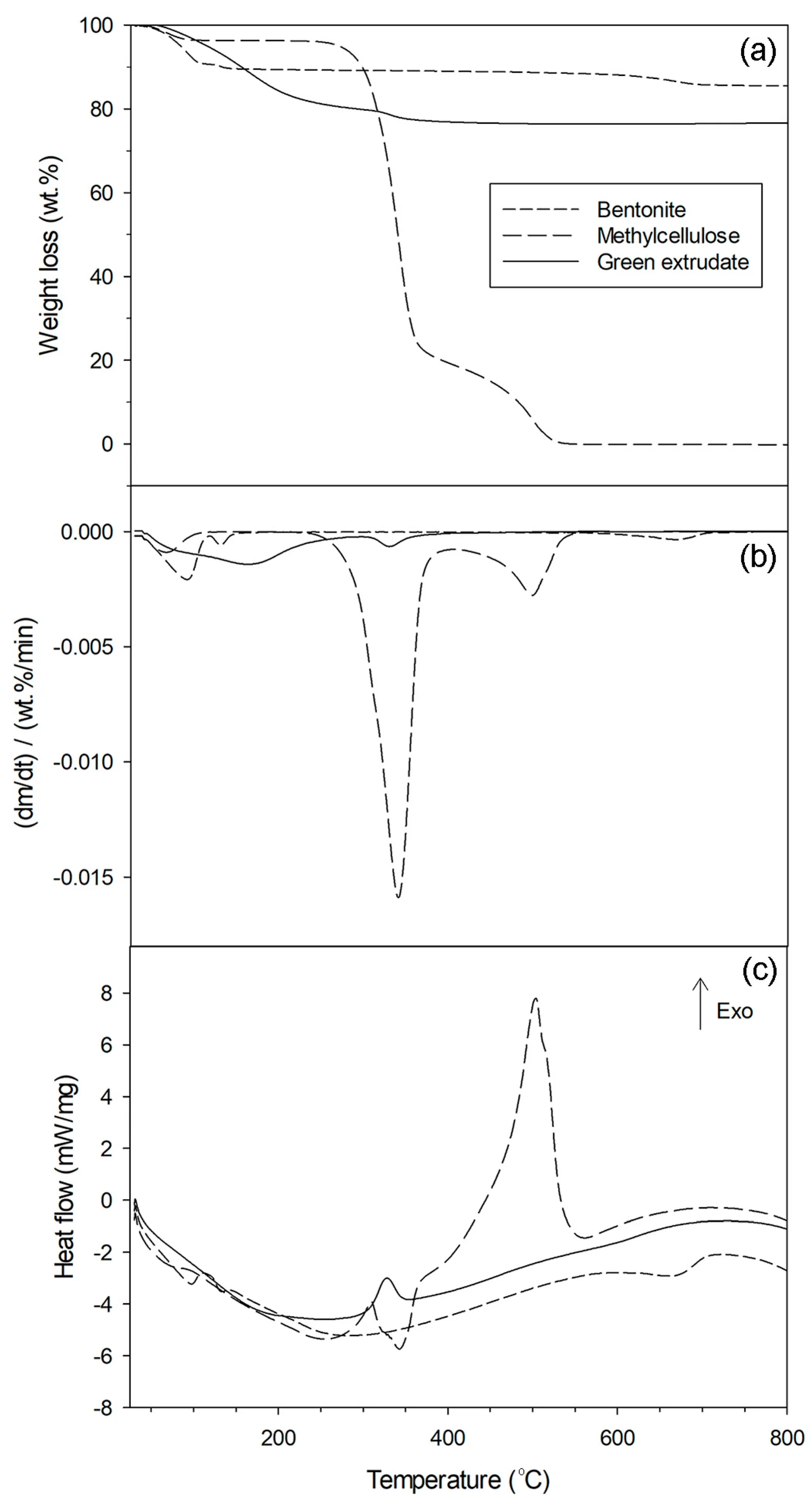
Thermogravimetric analysis of bentonite, methylcellulose, and green Extrudate 1: (**a**) weight loss; (**b**) differential weight loss; (**c**) heat flow.

**Figure 3:**
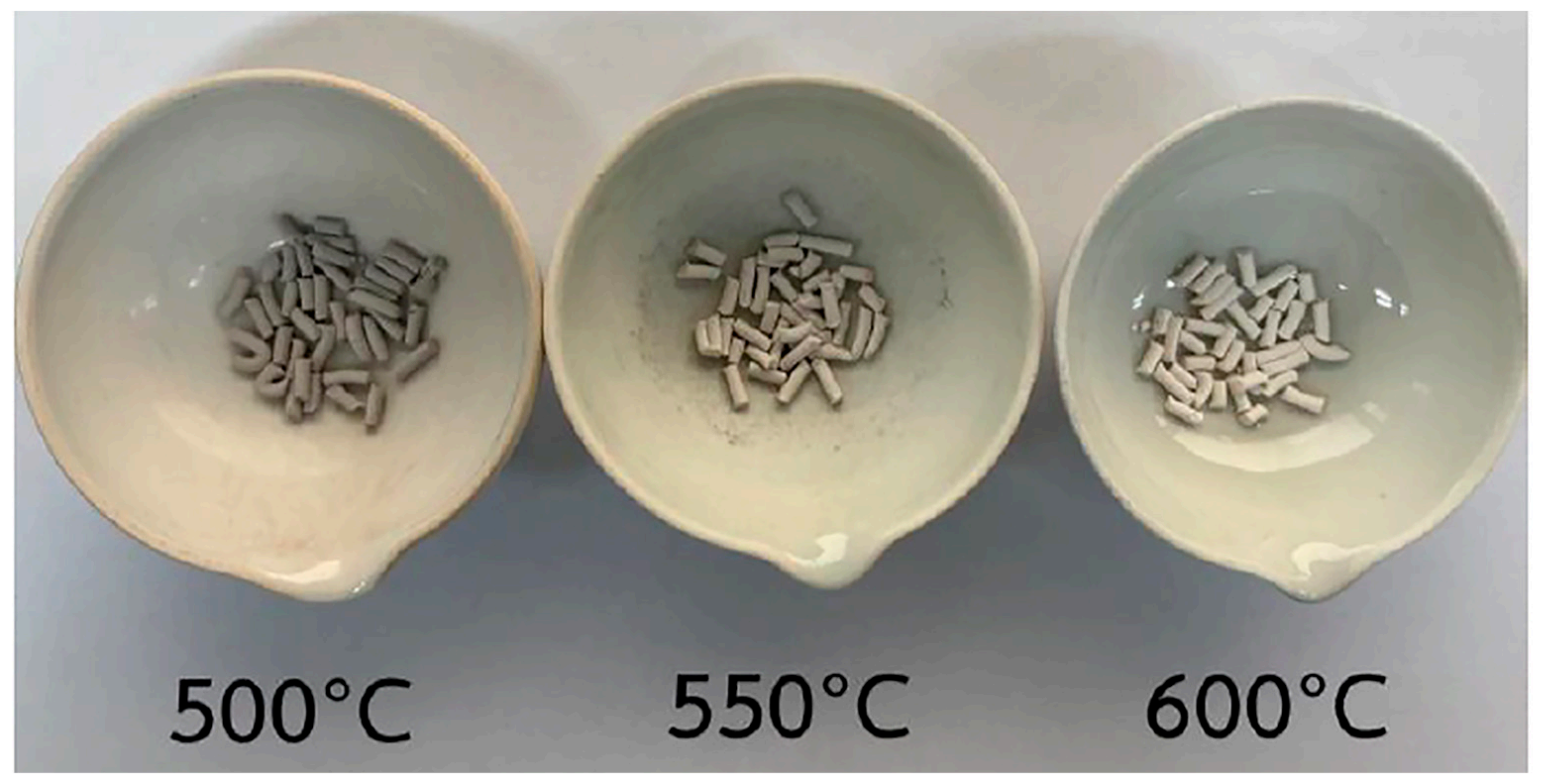
Physical appearance of Extrudate 1 at different calcination temperatures.

The NaX zeolite extrudates were successfully prepared in a cylindrical shape. The composition consisted of zeolite, bentonite, and methylcellulose. The green zeolite extrudate with a bentonite-to-zeolite weight ratio of 6:100 (Extrudate 1) was analyzed by TGA-DSC to determine mass losses and thermal behavior as a function of temperature. These findings provide a guideline for establishing the calcination program of the green extrudates. Figure 2 displays the thermogravimetric profiles of the green Extrudate 1, bentonite as the binder, and methylcellulose as the plasticizer. The TGA profile for bentonite exhibits three distinct mass losses, 30–119 °C (9 wt.%), 119–160 °C (1.5 wt.%), and 520–731 °C (4 wt.%), resulting in a total weight loss of 14.5 wt.%. All weight losses with desorption heat are assigned to water desorption from the bentonite. The weight loss at lower temperature (<160 °C) is from physically bound water, while the weight loss at higher temperature is from structural hydroxyl water from dehydroxylation. The TGA profile for methylcellulose occurs in three distinct mass losses, 30–102 °C (3.6 wt.%), 236–380 °C (75.1 wt.%), and 380–562 °C (21.1 wt.%), resulting in a total weight loss of 100 wt.%. The initial weight loss is attributed to water desorption, as indicated by the endothermic heat flow. Subsequent weight loss between 236 and 562 °C is assigned to the combustion of methylcellulose, which is characterized by a significant exothermic heat flow. In addition, the green Extrudate 1, composed of zeolite, bentonite, and methylcellulose, exhibits two primary weight loss stages. The first stage between 30 and 284 °C (20 wt.%) is attributed to the desorption of water from all three components. The second stage between 284 and 500 °C (4 wt.%) is assigned to the combustion of methylcellulose, as indicated by exothermic heat flow. Based on the results, a calcination temperature of 600 °C is determined to be sufficient for the complete removal of the methylcellulose from the green zeolite extrudates. The structural integrity of the ground Extrudate 1 is preserved after heat treatment, as evidenced by the characteristic NaX (FAU) diffraction patterns observed in the XRD analysis. In addition, the physical appearance of the extrudates after calcination treatment at 500, 550, and 600 °C was observed as shown in Figure 3. The final extrudate exhibits a dark gray color when calcined at a lower temperature of 500 °C. In contrast, samples treated at higher calcination temperatures of 550 and 600 °C display light gray and white colors, respectively. The transition to white color signifies the effective removal of methylcellulose. This observation is consistent with the TGA-DSC results. Therefore, calcining the extrudates at 600 °C is the optimal condition for the complete elimination of the methylcellulose organic compound.

**Figure 4:**
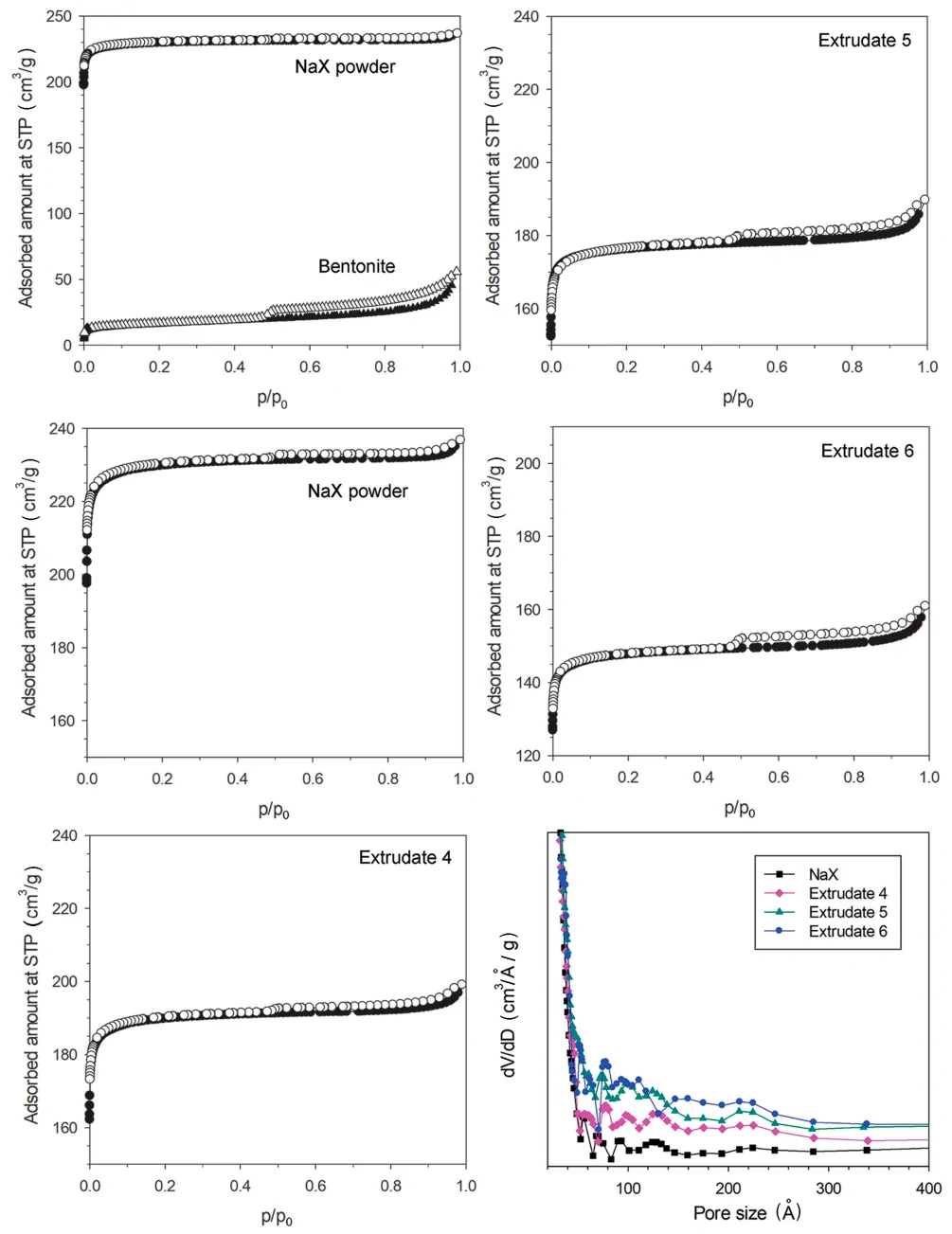
Isotherms of low-temperature N_2_-adsorption on NaX powder, bentonite, Extrudate 4, Extrudate 5, and Extrudate 6, and mesopore size distributions.

The bentonite binder content was varied relative to the zeolite powder, as summarized in Table 1. Specifically, bentonite-to-zeolite weight ratios of 6:100 (Extrudate 1), 7.2:100 (Extrudate 2), 8.4:100 (Extrudate 3), and 9.3:100 (Extrudate 4) were prepared, while maintaining constant amounts of methylcellulose and distilled water. Within this range, the paste maintained the necessary rheology for successful extrusion. The influence of the bentonite content on the textural properties becomes evident at a loading of 9.3:100 (Extrudate 4). At this content, the specific surface area and pore volume of the extrudate decreased by 12.5% and 15.8%, respectively, compared to the parent zeolite powder. The isotherms are presented in Figure 4, and the textural properties are summarized in Table 2. Extrudates with higher bentonite contents of 13.3:100 (Extrudate 5) and 26.7:100 (Extrudate 6) were also prepared. However, increasing the amount of bentonite in the formulation increases the paste’s dryness, thereby hindering the extrusion process and resulting in extrudates with significant surface cracking. This adversely affects the extrusion behavior of the rigid paste; consequently, the water content as the solvent and methylcellulose as the plasticizer must be adjusted until the mixture is sufficiently soft for extrusion.

The high bentonite contents of Extrudates 4, 5, and 6 lead to a reduction in specific surface area, micropore surface area, total pore volume, and micropore volume, and a slight increase in mesopore volume. The increase in mesopore volume can be confirmed by the presence of hysteresis loops in the isotherms at partial pressures between ca. 0.5 and 1 (Figure 4). In addition, Extrudates 5 and 6 exhibit reductions in specific surface areas of 22.7 and 35.9%, respectively, and a decrease in pore volumes of 19.7 and 32% relative to the parent NaX powder. The reduction in micropore volume is the primary contributor to the overall decrease in the textural properties of the extrudates. These results indicate that high bentonite content decreases the micropore surface area and volume by coating the zeolite crystals. This phenomenon can lead to partial pore-mouth plugging, which restricts access to the intracrystalline porous structure of the zeolite. The observed mesopore volume may originate from the bentonite fraction itself and from the intercrystalline voids created when zeolite particles are bound together by the bentonite binder. The mesopore size distribution (in Figure 4) reveals that new mesopores were generated after the shaping process. This provides clear evidence that these mesopores originate from intercrystalline voids created during extrusion, with the mesopore size distribution in the 7.5–24 nm range.

**Figure 5:**
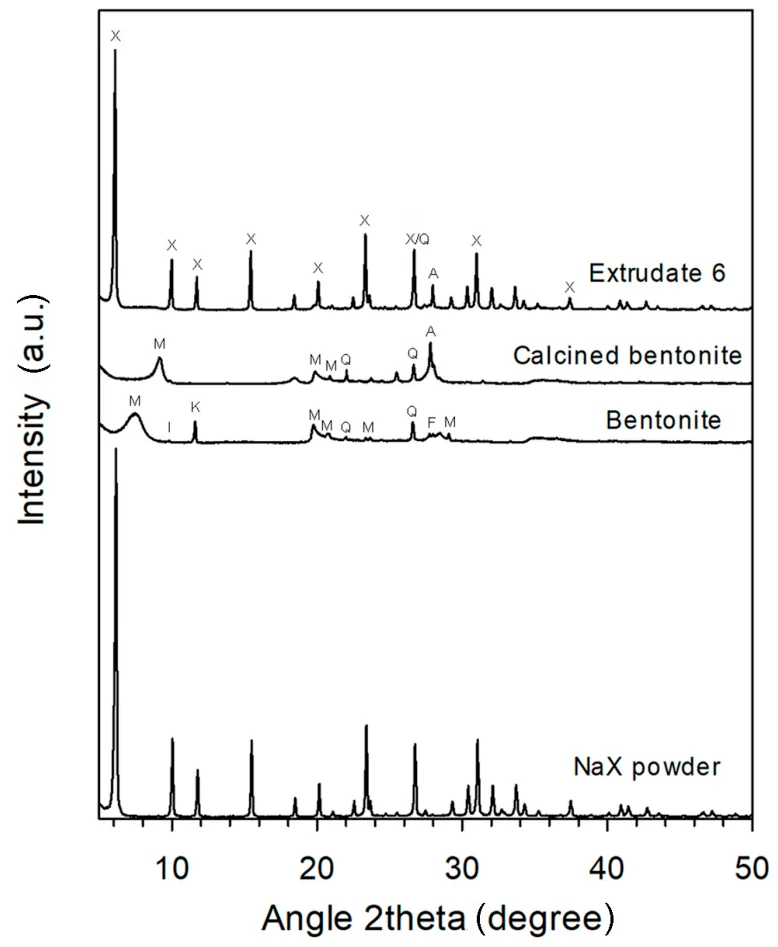
XRD patterns of Extrudate 6, NaX powder, bentonite, and calcined bentonite (X is Faujasite structure of NaX, M is montmorillonite, I is illite, K is kaolin, Q is quartz, F is feldspar, A is anorthoclase).

The XRD patterns of the original NaX powder, bentonite, calcined bentonite, and Extrudate 6 are shown in Figure 5. The crystalline structure of NaX in Extrudate 6 is maintained as the main phase, with the major peak positions at 2theta ≈ 6.09, 9.96, 11.77, 15.48, and 23.32° [17]. The slight shift of the peaks toward a lower 2theta indicates expansion of the crystal lattice spacing. This could be attributed to thermal dehydration by calcination treatment of Extrudate 6. Bentonite exhibits peak positions at 2theta ≈ 7.57, 19.77, 20.8, 29.09, and 33.36°, representing montmorillonite as the main phase [18]. Peaks at 2theta ≈ 20.8 and 26.63° are attributed to quartz [19], whereas peaks at 2theta ≈ 9.88° and 11.6° correspond to illite and kaolin, respectively [20,21]. Additionally, peaks at 2theta ≈ 28.02 and 28.45° are assigned to feldspar [22]. Quartz, kaolin, illite, and feldspar are impurity phases.

For bentonite, the broad characteristic peak of montmorillonite at 2theta ≈ 7.57° corresponds to the *d*_001_ basal reflection, representing interlayer spacing. According to Bragg’s law [23], the calculated interlayer *d*-spacing is 1.168 nm. After heat treatment under the same drying and calcination conditions as the extrudate, the first broad peak of calcined bentonite shifts to 2theta ≈ 9.19°. This shift indicates a decrease in the *d*-spacing to 0.962 nm due to structural dehydration. While quartz remains present, the kaolin structure collapses. A newly formed anorthoclase phase (an alkali-feldspar type) appears at 2theta ≈ 27.82 [24]. The XRD pattern of ground Extrudate 6 primarily consists of characteristic peaks of NaX, quartz, and the newly formed anorthoclase. The montmorillonite phase at the peak of 2theta ≈ 9.19° is completely absent, suggesting structural collapse under high temperatures in this environment. The breakdown of the montmorillonite lattice after heat treatment at 600 °C signifies thermal dehydroxylation and a permanent phase transformation into amorphous metabentonite. This can be supported by the TGA result (Figure 2) via indications of a small weight loss of 0.013% and a subtle endothermic behavior in the range of 590–660 °C of the extrudate. The metabentonite is a high-strength aluminosilicate material compared to the raw bentonite clay [25]. Therefore, the presence of the existing quartz phase, the metabentonite, and the emergence of new anorthoclase can create overall mechanical stability of the zeolite extrudates.

**Figure 6:**
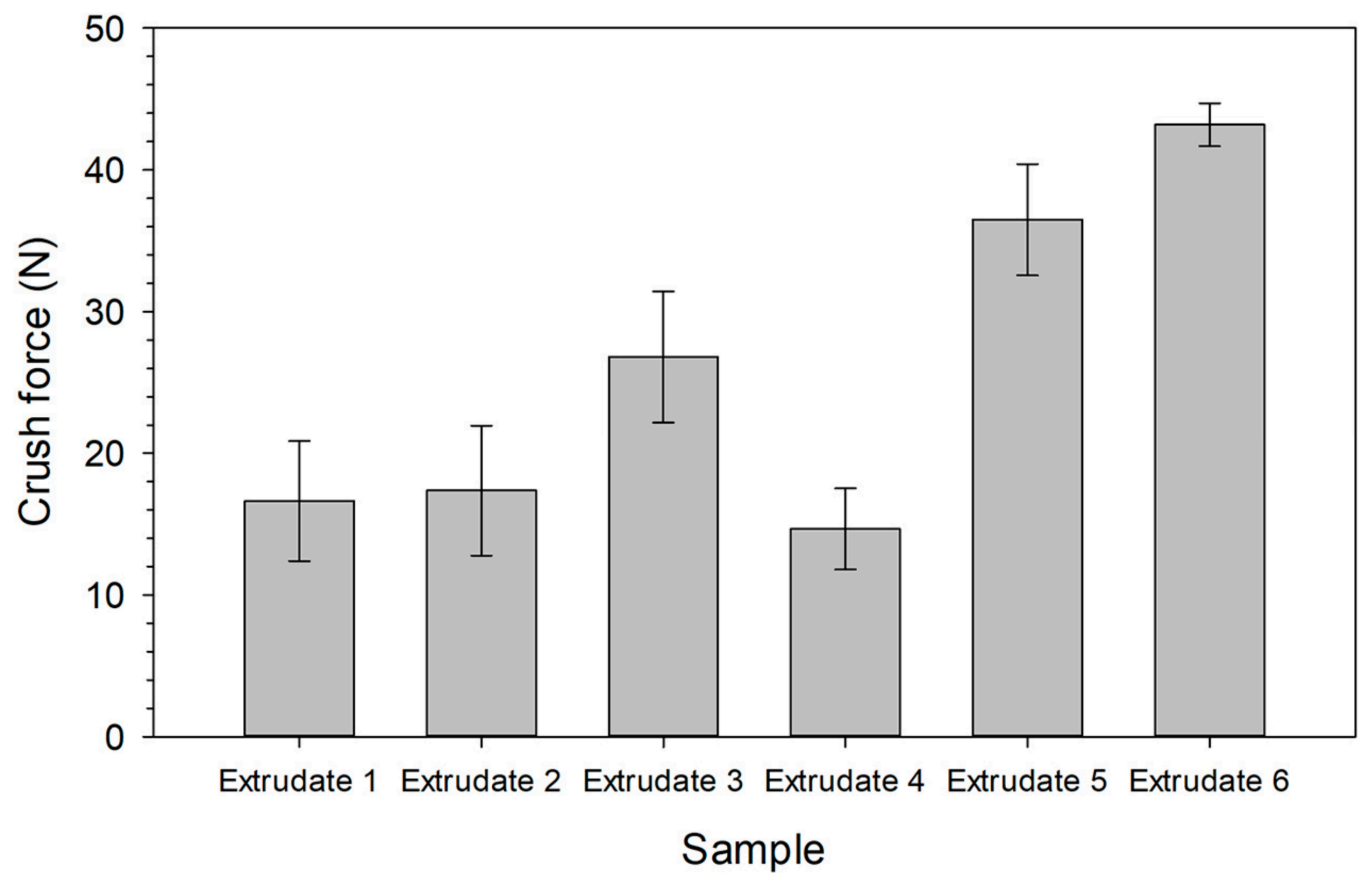
Crush forces of the extrudate samples. Bars represent the mean crush force, and the error bars indicate the standard deviation of the measurements.

**Figure 7:**
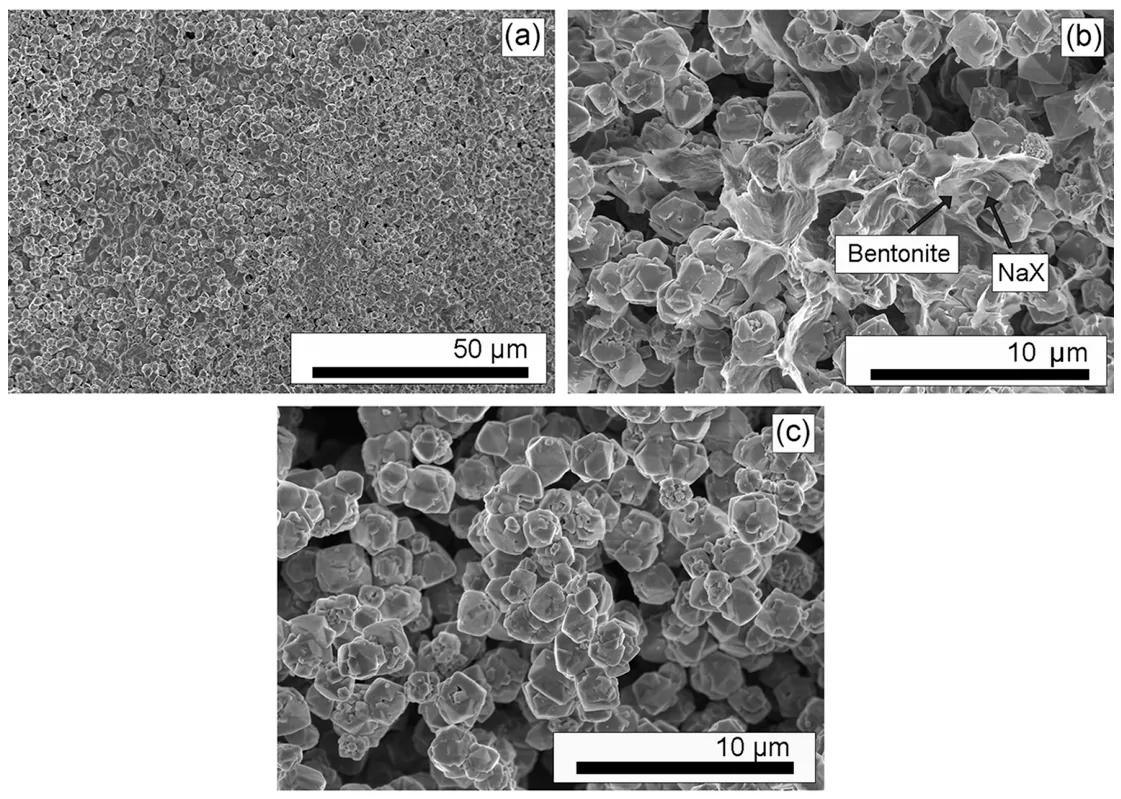
SEM images: (**a**) the cross-section of Extrudate 6 at 1000× magnification; (**b**) the cross-section of Extrudate 6 at 5000× magnification; (**c**) NaX powder at 5000× magnification.

The crush strength of the extrudates is a critical parameter for ensuring long-term mechanical durability during industrial use. The values are summarized in Table 3 and Figure 6. While the crush force increases from Extrudate 1 to 3, it drops for Extrudate 4. This decline could be attributed to a suboptimal composition for achieving mechanical strength. Specifically, as the bentonite content increases within an insufficient liquid solution, the extrudate surface tends to be rough and exhibits minor cracking. For Extrudates 5 and 6, the crush force continues to increase, reaching a maximum value of 43.19 N or a crush strength of 4.8 N/mm. The measured crush force of 43.19 N is comparable to the reported values for commercial zeolite NaX [26], confirming its suitability for industrial and large-scale utilization. The SEM pictures of the cracked Extrudate 6 allow for the characterization of the internal cross-section, as presented in Figure 7a,b. The presence of the bentonite binder coating the octahedral NaX crystals (ca. 2.5 μm) is evident. Compared to the parent NaX zeolite shown in Figure 7c, these results confirm the successful incorporation of the bentonite binder into the extrudates.

### 3.2. Dehumidification in Confined Space

**Figure 8:**
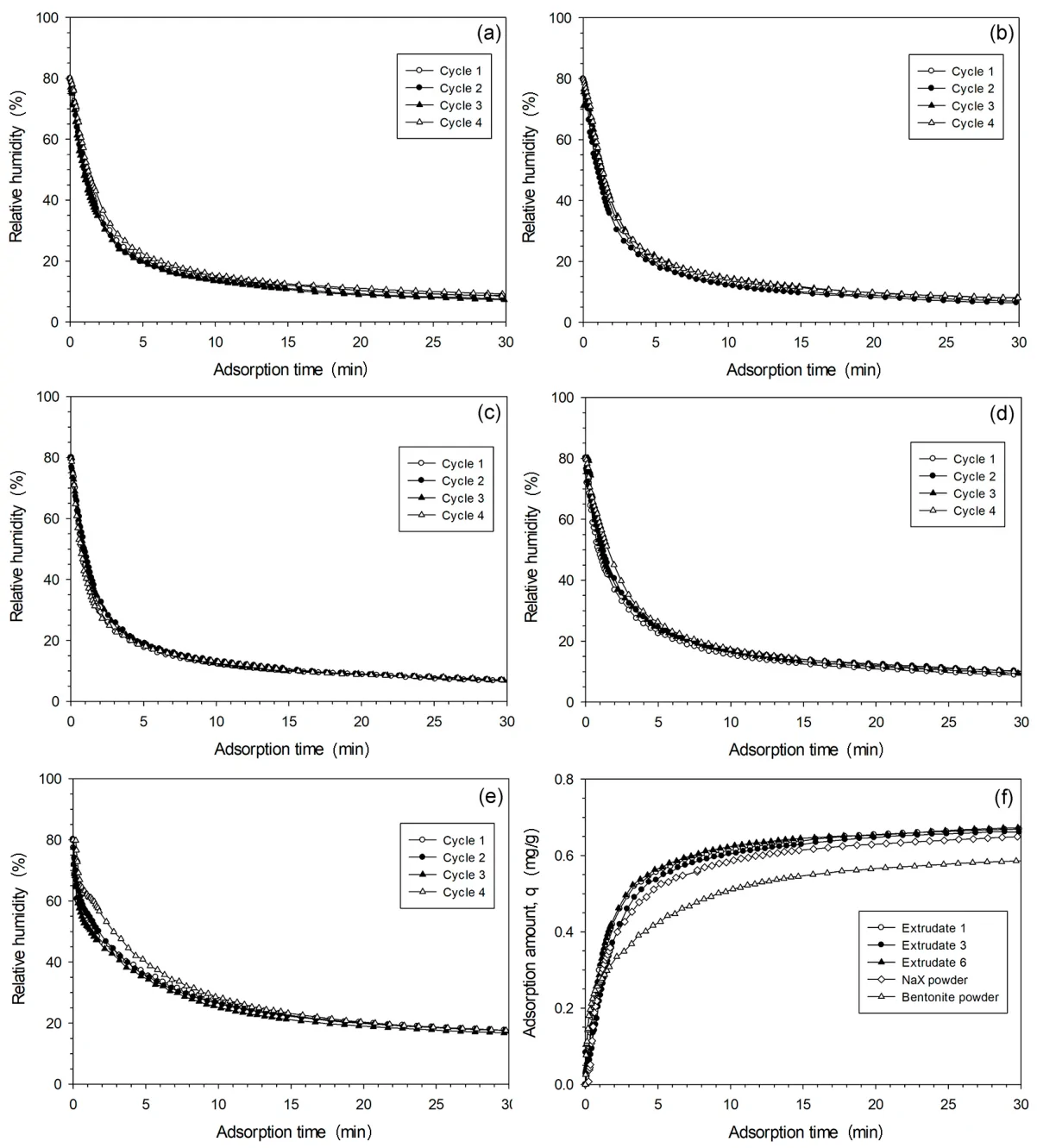
The relative humidity profiles of air in the climatic chamber tested with (**a**) Extrudate 1; (**b**) Extrudate 3; (**c**) Extrudate 6; (**d**) NaX powder; (**e**) bentonite powder; (**f**) the adsorption rate profiles for all samples.

Extrudate 1, 3, and 6 samples weighing 37.7 g were tested as dehumidifying adsorbents in the controlled climatic chamber. The relative humidity profiles for four cycles are presented in Figure 8a–c. Upon exposing the sample to the chamber, the relative humidity curves sharply decreased from 80% to approximately 30% within 2 min. This rapid decline is attributed to the maximum driving force between the initial ambient water vapor pressure and the active sites on the surface of the extrudates. Consequently, water vapor was adsorbed into the pore structure of the zeolite. The initial adsorption rates (Figure 8f) were determined from the slopes calculated over this one-minute interval. After 2 min, the adsorption rates gradually decreased and plateaued as the active sites became occupied and the water vapor pressure gradient between the air and the adsorbent surface narrowed. At approximately 25 min, the curve flattened out, indicating that extrudates reached quasi-equilibrium with the residual moisture in the air. Consequently, no further moisture uptake occurred. The final relative humidities, the adsorption amounts, and the adsorption rates are summarized in Table 4. The final relative humidities stabilized at quasi-equilibrium for Extrudates 1, 3, and 6 are 8, 7.3, and 6.8%, respectively. These correspond to water adsorption amounts of 25.07, 25.22, and 25.39 mg. Overall, they demonstrate strong affinity for moisture. Extrudate 6 shows the highest performance in terms of adsorption amount and adsorption kinetic rate, despite having the lowest fraction of zeolite to bentonite (78.93:21.07), and the lowest specific surface area and pore volume. This counterintuitive behavior can be attributed to an optimized pore hierarchy. The higher bentonite content facilitates a high-mechanical-strength matrix integrating with larger transport pores (e.g., mesopores). The water molecules can rapidly access and fully saturate the active zeolite sites deep within the extrudate core. This can be proved by a higher proportion of mesopores to micropores in Extrudate 6 (6.1: 93.9) when compared to those of Extrudates 1 and 3 (4.2: 95.8 and 4.1: 95.9). The optimal proportion can lead to better diffusion of the moisture to the active sites.

Regarding the adsorption of NaX powder, its adsorption amount and adsorption kinetic rate are lower than those of Extrudate 6, despite a higher net zeolite content (37.7 g vs. 29.76 g, respectively) (Figure 8d,f). This evidence clearly demonstrates the importance of shaping structure for enhancing the transport process of water molecules to the active sites located within the core of the extrudate. This prevents the dead zone and the agglomeration of the particles, which can retard the overall transport process. The pure bentonite powder can adsorb a lower water content of 21.88 mg (achieved at 17.23% relative humidity) and has a lower adsorption rate of 8.03 mg/min (Figure 8e and Table 4). Following the adsorption test, the spent samples were regenerated by calcination. This process effectively maintained the adsorption capacity for four operation cycles with comparable absorbed amounts in each cycle.

The amount of water adsorbed on the extrudate sample at time t can be calculated by subtracting the amount of water vapor remaining at that time from the amount of water vapor present in the chamber at the initial time. The amount of water vapor in the chamber can be determined from the relative humidity, temperature, Antoine equation, and humidity equation as shown in Equations (1)–(3) [27,28].

(1) $$\mathrm{RH}=\frac{p_{H2O}\cdot \;100}{p_{H2O}^{\prime }}$$

(2) $$\;\ln p_{H2O}^{\prime }=A-\frac{B}{\left(T+C\right)}$$

(3) $$H=\frac{m_{H2O}}{m_{\mathrm{air}}}\cdot 100=\frac{M_{H2O}\cdot p_{H2O}}{M_{\mathrm{air}}\left(P-{\;p}_{H2O}\right)}\cdot 100$$

where

*RH* is relative humidity (%);

*p_H2O_* is the vapor pressure of water (mmHg);

*p’_H2O_* is the saturation vapor pressure of water at that temperature (mmHg);

A, B, C are Antoine vapor pressure equation coefficients of 18.3036, 3816.44, −46.13, respectively, with vapor pressure in mmHg and temperature in Kelvins;

*H* is humidity (%);

*m_H2O_* is mass of water vapor (g);

*m_air_* is mass of air (g);

M_H2O_ is the molecular weight of water vapor (g/mol);

M_air_ is the molecular weight of air (g/mol);

*P* is total pressure (mmHg).

The adsorption profile of Extrudate 6 (cycle 3 as candidate) in Figure 8f will be fitted using two adsorption kinetic models of the pseudo-first-order model and the linear driving force (LDF) model. The differential rate equation for pseudo-first-order adsorption is presented in Equation (4).

(4) $$\frac{dq(t)}{dt}=k_{1}\;\left[q_{e}-q(t)\right]$$

where

*q(t)* is the amount of water vapor adsorbed in the adsorbent at time t (mg/g);

*q*_e_ is the amount of water vapor adsorbed in the adsorbent at equilibrium (mg/g);

*k*_1_ is the pseudo-first-order adsorption rate constant (1/min);

*t* is the adsorption time (min).

The pseudo-first-order adsorption kinetic model is an empirical model used to describe the rate of adsorption of adsorbate onto a solid adsorbent. Originally formulated by Lagergren [29], it describes that the rate of adsorption is directly proportional to the difference between the amount of adsorbate in the adsorbent at equilibrium and the amount of adsorbate in the adsorbent at a given time (or the unused sites) [30,31]. By integrating Equation (4) with the boundary condition of no water vapor adsorption at the start of the experiment (*q* = 0 at *t* = 0), Equation (5) and the linearization in Equation (6) are obtained.

(5) $$\ln\left[\frac{q_{e}-q(t)}{q_{e}}\right]={-k}_{1}\;t$$

(6) $$\ln\left[q_{e}-q(t)\right]={\ln q_{e}-k}_{1}\;t$$

Linear regression analysis using Equation (6) alongside the experimental equilibrium adsorption capacity (*q*_e_) of 0.714 mg/g yields a coefficient of determination (R^2^) of 0.8265. From this, the pseudo-first-order adsorption rate constant (*k*_1_) is determined to be 0.1283 1/min. Substituting these derived values back into Equation (6) produces Equations (7) and (8).

ln[0.714 − *q*(*t*)] = −0.3369 − 0.1283 *t* (7)

*q*(*t*) = 0.714 − e^−0.3369−0.1283 *t*^ (8)

Another adsorption kinetic model is the linear driving force (LDF) model. It was originally developed by Glueckauf and Coates [32], describing the adsorption rate based on the assumption that mass transfer within the adsorbent particle is controlled by internal intraparticle diffusion. The model was subsequently simplified to a linear rate equation for gas adsorption and separation processes [33]. This LDF model has been demonstrated to be a practical tool for describing the adsorption kinetics on heterogeneous solids, and its rate equation of single adsorbate from [33] is given in Equation (9).

(9) $$\frac{d\bar{C}(t)}{dt}=k_{L}\;\left[{\bar{C}}^{*}(t)-\bar{C}(t)\right]$$

where

$\bar{C}(t)$ is the average adsorbate concentration in the adsorbent particle at time *t* (mol/L);

${\bar{C}}^{*}(t)$ is the adsorbate concentration in the particle that would be in equilibrium with the instantaneous superincumbent gas phase partial pressure of the adsorbate and the adsorbent temperature at time *t* (mol/L);

*k*_L_ is the effective LDF mass transfer coefficient at adsorbate loading of *C* and temperature *T*.

In this case, in the batch adsorption with a constant volume, Equation (9) can be rearranged based on mass and adsorbent weight. Therefore,

(10) $$\frac{dq(t)}{dt}=k_{\mathrm{LDF}}\;\left[q^{*}(t)-q(t)\right]$$

where

*q(t)* is the amount of water vapor adsorbed in the adsorbent at time *t* (mg/g);

q^*^(t) is the amount of water vapor adsorbed in the adsorbent that would be in equilibrium with the instantaneous superincumbent gas phase partial pressure of the water vapor and the adsorbent temperature at time *t* (mg/g);

*k*_LDF_ is the effective LDF mass transfer coefficient at this adsorbate loading (*q*) and this temperature (1/min);

*t* is the adsorption time (min).

The main difference between Equation (10) and Equation (4) is that q*(t) is not constant like *q*_e_; instead, it varies with the remaining concentration within the confined space. Therefore,

(11) $$\frac{dq(t)}{dt}=k_{\mathrm{LDF}}\;\left[q^{*}\left(C(t)\right)-q(t)\right]$$

In a real confined space of finite volume, *V*, containing extrudate mass, *M*, the depletion of the water vapor concentration in the air, *C(t)*, must be balanced by the solid-phase accumulation, *q(t)*. The resulting material balance is expressed as

*V*[*C*_0_ − *C*(*t*)] = *M*[*q*(*t*) − *q*_0_] (12)

(13) $$C(t)=C_{0}-\left(\frac{M}{V}\right)q(t)$$

where

*q(t)* is the amount of water vapor adsorbed in the adsorbent at time *t* (mg/g);

*q*_0_ is the initial amount of water vapor adsorbed in the adsorbent (mg/g);

*C(t)* is the concentration of water vapor in the air in the confined space at time *t* (mg/L);

*C*_0_ is the initial concentration of water vapor in the confined space, 21.0276 (mg/L);

*M* is the mass of the extrudate, 37.7 (g);

*V* is the volume of the confined space, 1.317 (L).

The q^*^ can be derived from the Langmuir model and Freundlich model as shown in Equations (14) and (15), respectively.

(14) $$q^{*}(t)=\frac{\;q_{\max}\;\cdot K_{L}C(t)}{1+K_{L}C(t)}$$

q^*^(*t*) = *K*_F_*C*(*t*)^1/*n*^ (15)

where

*K*_L_ is the Langmuir adsorption constant (L/mg);

*q*_max_ is the maximum adsorption capacity (mg/g);

*K*_F_ is the Freundlich adsorption capacity constant (mg/g)(L/mg)^1/n^;

*n* is the adsorption intensity exponent (-).

Substitute Equation (13) into Equations (14) and (15), then substitute Equations (14) and (15) into Equation (11); Equations (16) and (17) are presented below.

(16) $$\frac{dq(t)}{dt}=k_{\mathrm{LDF}}\left[\frac{q_{\max}\cdot K_{L}\left(C_{0}-\frac{M}{V}q(t)\right)}{1+K_{L}\left(C_{0}-\frac{M}{V}q(t)\right)}-q(t)\right]\;$$

(17) $$\frac{dq(t)}{dt}=k_{\mathrm{LDF}}\;\left[K_{F}{\left(C_{0}-\frac{M}{V}q(t)\right)}^{1/n}-q(t)\right]$$

**Figure 9:**
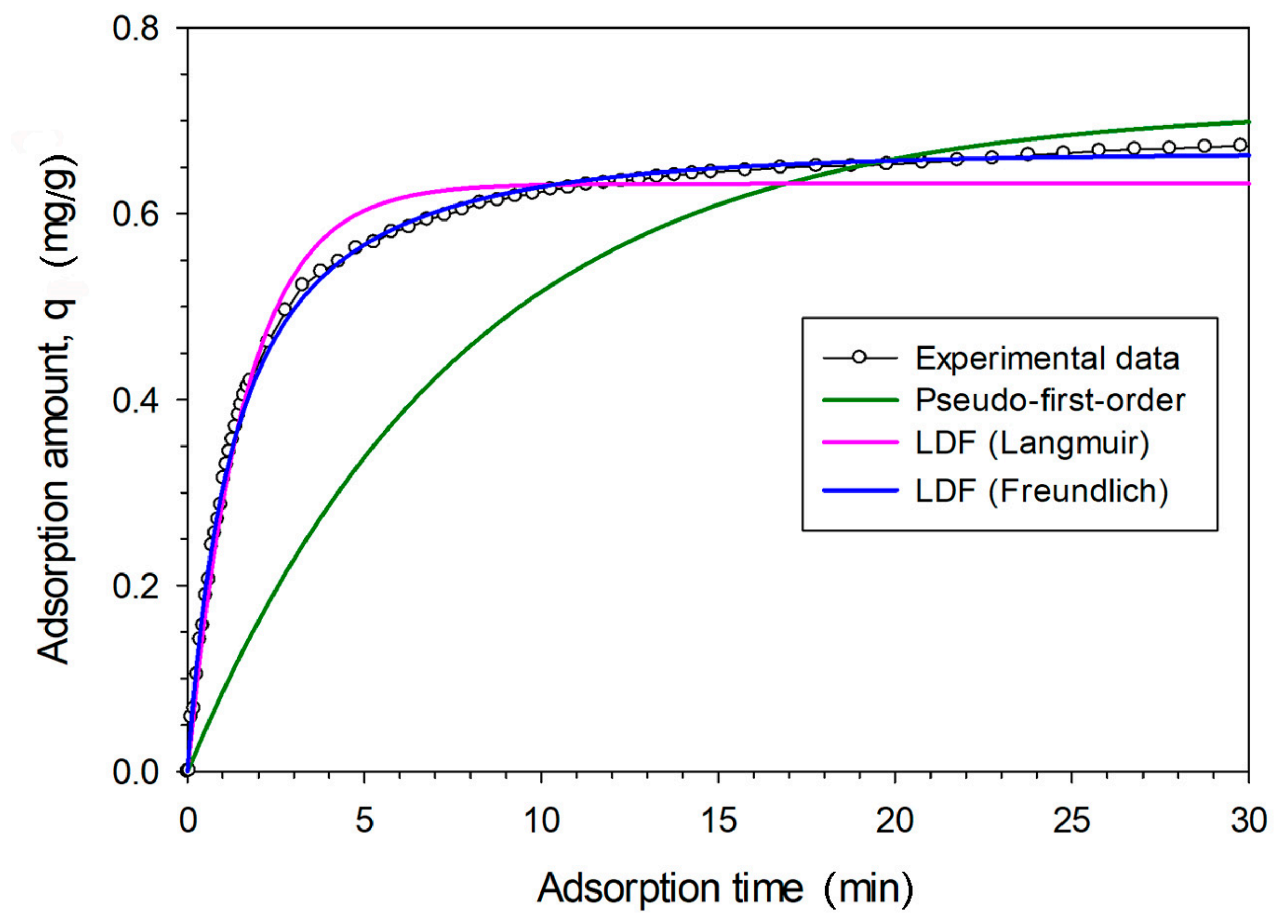
Adsorption kinetics of water vapor onto Extrudate 6 adsorbent fitted with pseudo-first-order model and linear driving force model coupled with Langmuir and Freundlich equations.

By substituting the known values, the parameter estimation is conducted by nonlinear ordinary differential equation curve fitting. For the Langmuir adsorption model, *k*_LDF_, *q*_max_, and *K*_L_ are 0.6060 1/min, 0.6379 mg/g, and 3.8335 × 10^44^ L/mg, respectively, with R^2^ of 0.9883. For the Freundlich adsorption model, *k*_LDF_, *n*, and *K*_F_ are 0.0073 1/min, 0.5020, and 0.1649 (mg/g)(L/mg)^1/n^, with R^2^ of 0.9979. The curve fittings of three cases to the experimental data are presented in Figure 9.

The results demonstrate that the linear driving force kinetic model combined with the Freundlich adsorption model provides an excellent fit to the experimental data, yielding the highest coefficient of determination (R^2^). This model yields an adsorption rate constant of 0.0073 1/min. Conversely, the pseudo-first-order model provides the poorest fit, exhibiting deviations particularly during the initial stage of the adsorption process.

Based on the model prediction of Equation (17) at equilibrium or with no change in adsorbed amount by time, the adsorbed water vapor capacity (*q*_e_) reaches 0.6643 mg/g, yielding an equilibrium moisture content in the air of 1.712 mg/g and a relative humidity of 7.9%. These values overestimate the empirical data recorded at the 2 h equilibrium adsorption (0.5 mg/g and 2.3%). However, such an empirical model and data are essential for determining the required dehumidification or drying time for packaging and laboratory applications, such as dry chambers. Consequently, it can accurately predict the time required for sensitive electronic components, pharmaceutical products, or chemical samples to reach a safe humidity threshold (typically < 10% relative humidity) after the container is sealed.

## 4. Conclusions

Cylindrical NaX zeolite extrudates are successfully prepared with a diameter of 2 mm and a length of 10 mm. The material retains a high specific surface area of 556 m^2^/g, a pore volume of 0.249 cm^3^/g, and a radial crush strength of 43.19 N (4.8 N/mm), confirming its suitability for practical, long-term applications. An optimal calcination protocol for the green NaX extrudates is by heating at 3 °C/min to 600 °C with a 3 h holding time in an air flow. This treatment ensures the removal of methylcellulose plasticizer while promoting the formation of anorthoclase and metabentonite phases. The presence of stable quartz (originating from bentonite), anorthoclase, and metabentonite in the zeolite extrudate is essential for enhancing the crush strength of the extrudate. Increasing the bentonite binder content from 9.3 to 26.7 g per 100 g of zeolite significantly influences the textural properties of the extrudate. Higher binder concentrations lead to a reduction in specific surface area and pore volume, particularly by disturbing the micropore fraction. Incorporating the bentonite binder slightly increases the mesopore volume and modifies the mesopore size distribution in the range of 7.5–24 nm, which can effectively improve mass transfer characteristics. The prepared NaX zeolite extrudates (26.7 g bentonite:100 g zeolite) can be effectively utilized as an adsorbent for moisture removal in a confined space. At an extrudate adsorbent to confined space volume ratio of 1 g: 34.9 cm^3^, the material reduces the relative humidity of moist air to 6.8% within 30 min. It exhibits an initial adsorption rate of 11.94 mg/min and a total adsorption capacity of 25.39 mg. The adsorption rate can be empirically fitted with the linear driving force (LDF) kinetic model coupled with the Freundlich adsorption model. Based on this model, the effective LDF mass transfer coefficient, kLDF, and the equilibrium adsorption capacity, q_e_, were determined to be 0.0073 1/min and 0.6443 mg/g, respectively. The prepared zeolite extrudates exhibit excellent cyclic stability, maintaining consistent adsorption performance over repeated use. The regeneration protocol is suggested by heating the material to 350 °C (at a ramp rate of 3 °C/min) under an N_2_ flow, followed by a 3 h holding time. This process effectively restores the adsorption capacity without any significant loss in performance over successive cycles.

## Figures and Tables

**Table 1:** Proportions by weight of the components in the extrudate paste.

Sample	Zeolite NaX	Bentonite	Methylcellulose	Distilled Water
Extrudate 1	100	6	3.05	99
Extrudate 2	100	7.2	3.05	99
Extrudate 3	100	8.4	3.05	99
Extrudate 4	100	9.3	3.05	99
Extrudate 5	100	13.3	3.71	133
Extrudate 6	100	26.7	5.94	244

**Table 2:** Textural properties of extrudates, NaX powder, and bentonite.

Sample	Surface Areas and Pore Volumes					
	Specific Surface Area (m^2^/g)	Micropore Surface Area (m^2^/g)	External Surface Area (m^2^/g)	Total Pore Volume (cm^3^/g)	Micropore Volume (cm^3^/g) (Pore < 2 nm)	Mesopore Volume (cm^3^/g) (Pore: 2–50 nm)
NaX powder	867	833	34	0.366	0.340	0.008
Bentonite	60	27	33	0.086	0.011	0.040
Calcined Extrudate 1	759	725	34	0.332	0.294	0.013
Calcined Extrudate 2	771	740	32	0.337	0.303	0.015
Calcined Extrudate 3	784	758	26	0.338	0.307	0.013
Calcined Extrudate 4	759	728	30	0.308	0.280	0.010
Calcined Extrudate 5	670	641	28	0.294	0.260	0.013
Calcined Extrudate 6	556	527	29	0.249	0.216	0.014

**Table 3:** Average crush forces and radial crush strength of zeolite extrudates.

Sample	Crush Force (N)	Radial Crush Strength (N/mm)
Extrudate 1	16.62	1.85
Extrudate 2	17.37	1.93
Extrudate 3	26.82	2.98
Extrudate 4	14.65	1.63
Extrudate 5	36.50	4.06
Extrudate 6	43.19	4.80

**Table 4:** Adsorption test results of final relative humidities, water adsorption amounts, and adsorption rates of samples over 4 cycles.

Sample	Cycle Number	Final Relative Humidity (%)	Water Adsorption Amount, Q_ads_ (mg)	Adsorption Rate (mg/min)
Extrudate 1	1	8.4	24.86	9.37
	2	7.3	25.23	10.36
	3	7.1	25.30	11.28
	4	9	24.89	8.89
Extrudate 3	1	6.9	25.37	8.54
	2	6.4	25.53	10.19
	3	8.1	24.96	8.39
	4	7.9	25.03	8.34
Extrudate 6	1	6.7	25.43	12.38
	2	6.9	25.37	10.62
	3	6.8	25.40	11.49
	4	6.9	25.37	13.28
NaX powder	1	8.8	24.72	11.11
	2	10.1	24.35	8.95
	3	9.4	24.52	10
	4	10.6	24.35	7.60
Bentonite	1	17.5	21.84	8.86
powder	2	17.4	21.73	7.49
	3	16.6	22.15	9.46
	4	17.4	21.80	6.29

## Data Availability

The original contributions presented in this study are included in the article. Further inquiries can be directed to the corresponding author.
